# LeMeDISCO is a computational method for large-scale prediction & molecular interpretation of disease comorbidity

**DOI:** 10.1038/s42003-022-03816-9

**Published:** 2022-08-25

**Authors:** Courtney Astore, Hongyi Zhou, Bartosz Ilkowski, Jessica Forness, Jeffrey Skolnick

**Affiliations:** Center for the Study of Systems Biology, School of Biological Sciences, Georgia Institute of Technology, Atlanta, GA 30332 USA

**Keywords:** Computational biology and bioinformatics, Translational research

## Abstract

To understand the origin of disease comorbidity and to identify the essential proteins and pathways underlying comorbid diseases, we developed **LeMeDISCO** (**L**arge-Scal**e M**olecular Int**e**rpretation of **Dis**ease **Co**morbidity), an algorithm that predicts disease comorbidities from shared mode of action proteins predicted by the artificial intelligence-based **MEDICASCY** algorithm. **LeMeDISCO** was applied to predict the occurrence of comorbid diseases for 3608 distinct diseases. Benchmarking shows that **LeMeDISCO** has much better comorbidity recall than the two molecular methods XD-score (44.5% vs. 6.4%) and the S_AB_ score (68.6% vs. 8.0%). Its performance is somewhat comparable to the phenotype method-based Symptom Similarity Score, 63.7% vs. 100%, but **LeMeDISCO** works for far more cases and its large comorbidity recall is attributed to shared proteins that can help provide an understanding of the molecular mechanism(s) underlying disease comorbidity. The **LeMeDISCO** web server is available for academic users at: http://sites.gatech.edu/cssb/LeMeDISCO.

## Abstract (web summary)

LeMeDISCO predicts disease comorbidities and the proteins and pathways responsible for comorbidity, validated in ovarian cancer and coronary heart disease.

## Introduction

Of the total of 3,634,743 disease pairs involving 13,034 distinct diseases in clinical data from 13,039,018 individuals^1^, 78.8% involving all 13,034 diseases have a larger than random probability that they co-occur in one individual. Disease comorbidity, the co-occurrence of distinct diseases in one individual, is an interesting medical phenomenon, and it is important to understand their molecular origins. For example, rheumatoid arthritis, autoimmune thyroiditis, and insulin-dependent diabetes mellitus co-occur, but rheumatoid arthritis and multiple sclerosis do not^2^. Previously, there have been several efforts to investigate the molecular features responsible for human disease comorbidities^3–9^. Some studies focused on particular subsets of diseases^4^ or ethnic groups, while others investigated the entire human disease network^5–8^. For example, ref. ^6^ applied text mining to search the literature for disease-symptom associations. They then predicted the entire human disease–disease network based on a calculated symptom similarity score. While this approach covers many human diseases, it relies on prior knowledge of symptomatic information; this limits its disease coverage and only explains one phenotype (disease) by another phenotype (symptom). ref. ^7^ utilized known disease–gene associations from GWAS^10^ and OMIM combined with a protein–protein interaction network to identify connected disease–gene clusters or modules. Another study also utilized known disease–gene associations and protein–protein interaction networks to characterize disease–disease relationships without requiring gene clusters^8^; thus, its disease coverage is better than in ref. ^7^. These studies that used known disease–gene associations are limited by data availability. Indeed, only a small fraction of diseases have known associated genes. For example, ref. ^8^ only covers 1022 of the 8043 diseases in the Disease Ontology database^11^, with just 6594 pairs of diseases having a non-zero number of shared genes. Similarly, ref. ^7^ found that about 59% of 44,551 disease pairs do not share genes and their relationship cannot be resolved based on the shared gene hypothesis. The effect of possibly missed proteins arising from both direct and indirect protein–protein interactions with known interacting proteins are accounted for by the network propagation method in the XD-score^8^ or by the disease module and network distance of the S_AB_ score^7^. However, those scores only marginally improve the recall rate of disease pairs that are clinically comorbid compared to that of shared genes in their methods (<10% recall rate by both the XD-score and S_AB_ score).

To address these limitations of existing studies, we developed **LeMeDISCO**, which extends our recently developed **MEDICASCY** machine learning approach^12^ for predicting disease indications and mode of action (MOA) proteins (as well as small molecule drug side effects and efficacy) to predict disease comorbidities and the proteins and pathways responsible for their comorbidity. **LeMeDISCO** covers 6.5 million pairs of diseases compared to 97,666 pairs by the XD-score^8^, 44,551 pairs by the S_AB_ score^7^, and 133,107 pairs by the Symptom Similarity Score^6^. Assuming that the most enriched comorbid proteins are responsible for disease comorbidity, we determine the most frequent comorbidity enriched MOA proteins. These proteins are then employed in pathway analysis^13^. As examples, we predict the comorbid diseases, comorbidity enriched MOA proteins, and pathways associated with coronary artery disease (CAD) and ovarian cancer (OC). We note that recently machine learning (ML) methods have been successfully employed in numerous areas of biology^12,14–16^. However, due to MLs “black box” nature, it is not easy to trace back the biological meaning of the predictions and the molecular origin(s) of disease comorbidity. Thus, as in previous works^6–8^, we adopt an explicit score that provides a set of common proteins responsible for comorbidity predictions.

## Results

### Benchmarking results of LeMeDISCO

To assess its relative performance, we compared the results of **LeMeDISCO** to three other methods, the XD-score^8^, the S_AB_ score^7^, and the Symptom Similarity Score^6^. The XD-score was calculated as described in ref. ^8^: Using known disease–gene associations to create a vector representation of the disease by setting 1 for all associated genes and 0 for all others; then the vector was iteratively updated based on the Random Walk with Restart (RWR) algorithm, with a restart probability of *p* = 0.9 by using the STRING network database. Finally, the XD-score quantifying the relation of two diseases is defined using the updated vectors of two diseases. NG is the number of shared genes between disease pairs^8^. The *S*_*AB*_ score, a protein–protein network-based separation of a disease pair calculated from known disease–gene associations is defined as S_AB_ = <d_AB_>− (<d_AA_> +<d_BB_>)/2, where S_AB_ compares the shortest distances between proteins within each disease A & B^7^, <d_AA_> and <d_BB_> , to the shortest distances <d_AB_ > between A-B protein pairs^7^. The Symptom Similarity Score was obtained by large scale text mining of the literature for disease-symptom relations represented as a vector, with the similarity score defined as the cosine similarity of the respective vectors^6^. In this work, a J-score for disease similarity is defined as the Jaccard index^17^ of two diseases (see Method for details). The disease–disease relations of benchmarking data from Medicare insurance databases are quantified by their relative risk (RR) and φ**-**score (see Methods section for details)^1^. The relative risk RR is defined in Eq. 4a and is the probability that two diseases occur in a single individual relative to random. The φ**-**score is the Pearson’s correlation for binary variables and is defined in Eq. 4b. Diseases in this work are represented by DOID numbers from the Human Disease Ontology database^11^, and they are in clinical data usually denoted by ICD-9 or ICD-10 classifications^18^ or their Medical subject headings (MeSH) names^19^.

Table 1 summarizes the results. We define a true positive comorbidity pair when their clinic log(RR) >0, a predicted positive when XD-score >0, *S*_*AB*_ score <0, or the Symptom Similarity Score >0.1 and *q* value <0.05 for our J-score. Recall is defined as (the number of true positives having score > cutoff or < cutoff for *S*_*AB*_ score)/(total number of true positives). We emphasize that in calculating recall, the cutoffs are suggested by the respective work as being either biologically meaningful^7,8^ or statistically significant^6^. In addition to Pearson’s correlation coefficient (c.c.), recall and precision, the cutoff independent measures area under the receiver operating characteristic (AUROC) and the area under the precision-recall curve (AUPRC) are also compared.

**Table 1:** Comparison of LeMeDISCO’s J-score with the XD-score, NG, *S*_*AB*_ score and Symptom Similarity Score for correlations with comorbidity quantified by the log(RR) score, φ**-**score, and recall^a^.

	Log(RR) score	φ-score	Recall	Precision	AUROC	AUPRC
*191,966 disease pairs*^*b*^						
LeMeDISCO	**0.116 (0.0)**	**0.090 (0.0)**	**37.1%**	**77.2%**	**0.528**	**0.780**
Permute drug–protein [*p* value]	0.050 ± 0.004 (0.0) [1.3 × 10^−54^]	0.060 ± 0.004 (0.0) [2.1 × 10^−12^]	8.8 ± 0.7% [2.0 × 10^−315^]	74.7 ± 1.3%[0.027]	0.495 ± 0.006 [3.5 × 10^−9^]	0.755 ± 0.004 [4.9 × 10^−11^]
Permute drug–disease [*p* value]	0.0026 ± 0.0056 (0.24) [4.0 × 10^−92^]	0.0029 ± 0.0075 (0.19) [1.2 × 10^−31^]	0.0137 ± 0.0828% [0.0]	54.3 ± 46.5% [0.31]	0.500 ± 0.001 [1.7 × 10^−112^]	0.757 ± 0.0006 [2.3 × 10^−294^]
*29,658 pairs*^*c*^						
LeMeDISCO	**0.146 (0.0)**	**0.106 (0.0)**	**44.5%**	**80.6%**	**0.531**	**0.812**
XD-score^8^	0.042 (2.8 × 10^−13^)	0.071 (9.7 × 10^−35^)	6.4%	77.8%	0.510	0.801
NG^d^	0.0047 (0.42)	0.053 (6.6 × 10^−20^)	—	—	—	—
*943 disease pairs*^*e*^						
LeMeDISCO	**0.0986 (0.0024)**	**0.0886 (0.0065)**	**68.6%**	77.7%	**0.529**	**0.798**
*S*_*AB*_ score^7^	−0.0620 (0.057)	−0.0413 (0.205)	8.0%	**85.3%**	0.434	0.761
*2621 disease pairs*^*f*^						
LeMeDISCO	0.140 (5.2 × 10^−13^)	0.135 (3.8 × 10^−12^)	63.7%	79.3%	0.512	0.814
Symptom similarity^6^	**0.322 (0.0)**	**0.194 (1.4** × **10**^**−23**^**)**	**100%**	**79.6%**	**0.587**	**0.856**

^a^Numbers in parentheses “()” are the *p* values of the corresponding correlation. Bold indicates the best results for the given dataset. For the permutations of drug–protein and drug–disease relationships, the average ± standard deviation of 100 runs with different random seeds was given, the number in parenthesis “[]” is the *p* value converted from the *z*-score = (LeMeDISCO value-average)/standard deviation to characterize the statistical significance of the difference between LeMeDISCO and permutation tests.

^b^Mapping the DOID IDs from the human DO database to ICD-9 IDs of ref. ^1^, gives a set of 191,966 disease pairs.

^c^Mapped the ICD-9 disease code to our DOID of DO and obtained a consensus subset of 29,658 disease pairs from Table 1’s dataset of 97,665 disease pairs in ref. ^8^.

^d^NG is the number of shared genes between disease pairs in ref. ^8^.

^e^Consensus set of 943 disease pairs from the dataset of ref. ^7^ and our dataset of 191,966.

^f^A consensus dataset of 2621 disease pairs was obtained from their Supplementary dataset 4 of ref. ^6^ compared to our set of 191,966 pairs.

Mapping the DOIDs to the ICD-9 ID classifications of ref. ^1^, excluding easy pairs when in the MEDICASCY library two diseases have $\frac{{shared\; \#\; of\; efficious\; drugs}}{\sqrt{{\#\; disease}1{efficious\; drugs}\times {\#\; disease}2{efficious\; drugs}}} \; > \; 0.9$, we obtain 191,966 disease pairs for use in **LeMeDISCO** benchmarking. All Pearson’s correlations of the J-score with the log(RR) score (c.c. = 0.116, *p* value = 0) and φ**-**score (c.c. = 0.090, *p* value = 0) are statistically significant (*p* value <0.05). The recall rate of J-score for this large set is 37.1%, and the AUROC of 0.528 is well better than random of 0.5.

The Permute drug–protein test has an average ± standard deviation from 100 runs 1958.6 ± 144.9 (54.3%) for diseases with non-zero MOA proteins. We note there are still significant correlations, though the absolute c.c. drops from 0.116 to 0.050 (*p* value = 0.0) for log(RR) and from 0.090 to 0.060 (*p* value = 0.0) for the φ**-**score, and the recall drops from 37.1% of true relationships to 8.8% due to that the number of diseases having correctly assigned MOA proteins drops to around half. All other measures are also worse. The *p* values of the difference between **LeMeDISCO** and this permutation test are significant for all measures (<0.05).

On average, the Permute drug–disease test only has 55.09 (1.5%) diseases with non-zero MOAs. Its average recall of 0.0137% is much worse than that of the Permute drug–protein test because it loses the correct connections between diseases and proteins. Correlations with both log(RR) (c.c. = 0.0026, *p* value = 0.24) and the φ**-**score (c.c. = 0.0029, *p* value = 0.19) are insignificant. Except for precision, the *p* values of the difference between **LeMeDISCO** and this permutation test are all very significant (well below 0.05). The 54.3% average precision is due to its very few predictions (average only ~26). With these few predictions, a random selection of 26 pairs from the 191,966 (75.6% are true positives defined as log(RR) >0) will have a probability of $\mathop{\sum }\nolimits_{k=14}^{26}{C}_{26}^{k}$ × 0.756^k^ × 0.244^26-k^ = 0.996 of having greater than 54.3% precision. This means the precision is not better than random selection.

To understand the significant difference between the Permute drug–protein and the Permute drug–disease tests, we note that MEDICASCY predicts drug–disease pairs based on two components: One uses the drug’s chemical structure to learn the indications of a drug from those drugs with similar structure. This component is insensitive to whether the drugs’ protein targets change. The other depends on the drug’s protein targets. In the Permute drug–protein test, a permuted drug–protein relation will randomly change the drug’s protein targets to another drug’s. MEDICASCY was applied after the permutation to ensure correct drug–disease relations. Thus, MEDICASCY’s prediction of drug–disease relations still has information from the permuted drug–protein relation and the disease-(through permuted drug)–protein relations are not completely lost. This actually reflects the fact that there are a subset of proteins that occur in many diseases and permuting the drug–protein relationship for this subset does not change the identification of proteins in a given disease. On the other hand, the permuted drug–disease test completely destroys the mapping of the protein (through the drug) to disease.

To compare **LeMeDISCO**’s J-score to the XD-score, we mapped their ICD-9 disease code to the DOIDs and obtained a subset of 29,658 pairs from their dataset of 97,665 pairs^8^. As shown in Supplementary Fig. 1 and Table 1, the XD-score has a c.c. of 0.042 (*p* value = 2.8 × 10^−13^) with log(RR) and c.c. = 0.071 (*p* value = 9.7 × 10^−35^) with the φ**-**score. Their NG score (the number of shared genes) essentially has no significant correlation with log(RR) with a c.c. of 0.0047 (*p* value = 0.42) and only shows a correlation of 0.053 (*p* value = 6.6 × 10^−20^) with the φ**-**score. The J-score has much better correlations: c.c. = 0.146 (*p* value = 0.0) with log(RR), 0.106 (*p* value = 0.0) with the φ**-**score. The recall rate of the J-score is 44.5% compared to 6.4% for the XD-score. J-score’s precision (80.6 vs. 77.8%), AUROC (0.531 vs. 0.510), AUPRC (0.812 vs. 0.801) are all better. Supplementary Fig. 1 shows distinct patterns of J-score and XD-score. The data points of the XD-score are mostly concentrated at an XD-score = 0, whereas those of the J-score spread across the full range of 0–1.

For comparison with the *S*_*AB*_ score^7^, the MeSH^19^ disease names were mapped to their DOIDs. A consensus set of 943 disease pairs from their dataset and ours was obtained. As shown in Supplementary Fig. 2 and Table 1, compared to *S*_*AB*_^7^, for the 947 disease pairs, **LeMeDISCO**’s J-score has a c.c. = 0.0986 (*p* value = 0.0024) with log(RR) and a c.c. = 0.0886 (*p* value = 0.0065) with the φ**-**score that are both better than those of the *S*_*AB*_ score with a c.c. = −0.0620 (*p* value = 0.057) with log(RR) and c.c. = −0.0413(*p* value = 0.205) with the φ**-**score; both are insignificant. The recall rate of the J-score is 68.6% and is an order of magnitude better than the 8.0% by the *S*_*AB*_ score when defining comorbid pairs when the *S*_*AB*_ score <0; that is for a biologically meaningful disease–disease relationship^7^. J-score has AUROC = 0.531 compared to *S*_*AB*_ score’s 0.434 that is even worse than random value 0.5 because its dominant *S*_*AB*_ score >0 region is worse than random. The J-score also has a better AUPRC (0.798 vs. 0.761). However, J-score’s precision (77.7% vs. 85.3%) is slightly worse. Supplementary Fig. 2 shows that the data points of the *S*_*AB*_ score are concentrated in the region *S*_*AB*_ >0, whereas those of the J-score spread over the 0–1 region.

Next, a common dataset of 2621 disease pairs was obtained for comparison with the Symptom Similarity Score^6^. As shown in Supplementary Fig. 3 and Table 1, the Symptom Similarity Score has better correlations of 0.322 (*p* value = 0.0) than 0.140 (*p* value = 5.2 × 10^−13^) by the J-score for the log(RR) and 0.194 (*p* value = 1.4 × 10^−23^) than 0.135 (*p* value = 3.8 × 10^−12^) by the J-score for the φ**-**score. It also has better recall (100 vs. 63.7%), AUROC (0.587 vs. 0.512) and AUPRC (0.856 vs. 0.814). However, the Symptom Similarity Score only explains the relationship of one phenotype (symptom) to another phenotype (disease). Nevertheless, all correlations of the J-score are statistically significant. The J-score’s precision is almost identical to that of the Symptom Similarity Score (79.3 vs. 79.6%). We note that Supplementary Figs. 3, 4 show very similar patterns of the J-score and Symptom Similarity Score.

### MEDICASCY based MOA protein prediction

**Fig. 1:**
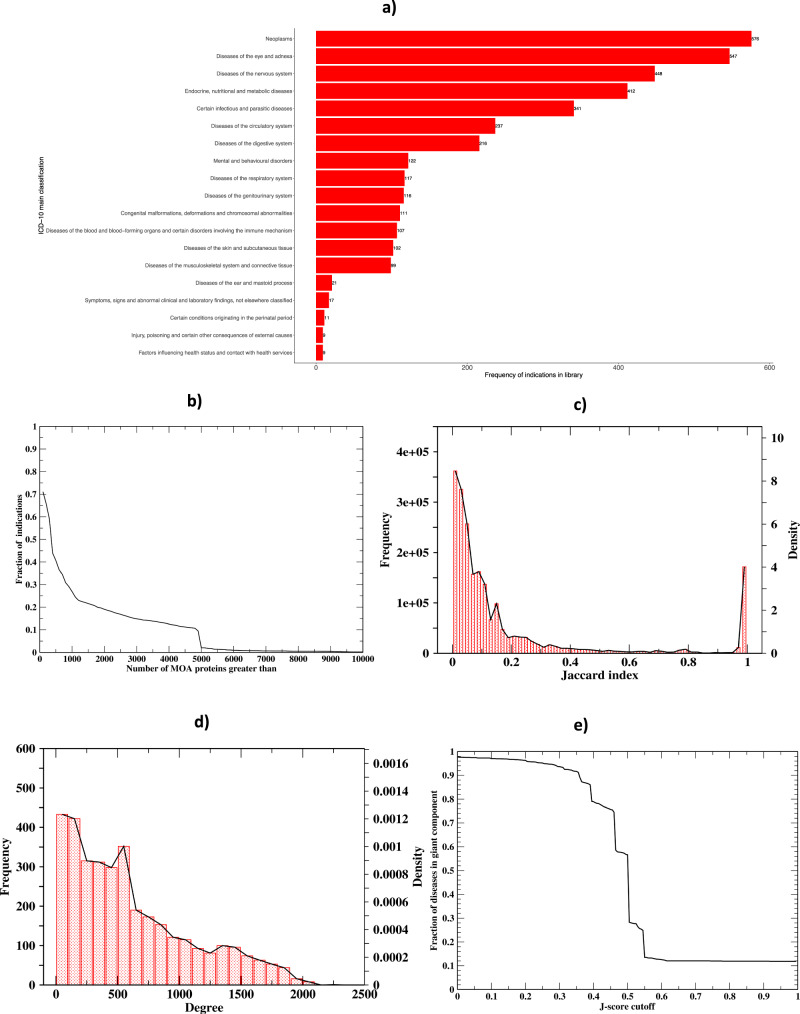
Summary results for 3608 distinct diseases. **a** ICD-10 main classification coverage across the 3608 diseases. Some diseases are found in multiple groups; they were counted in each group with which they are associated. **b** Histogram of the number of MOAs. **c** Frequency (bin size 0.02) and density of the J-score for the ~2 million significant (*q* value <0.05), non-redundant disease pairs. **d** Frequency (bin size = 100) and density of the degree (number of edges) of each disease (node). **e** Fraction of diseases in the giant component of disease–disease network versus the J-score cutoff.

The ICD-10 main classification coverage of the 3608 diseases is shown in Fig. 1a. We first examine the number of predicted MOA proteins per indication from **MEDICASCY**^12^. Using a *q* value cutoff of 0.05 and including protein isoforms, the average (median) number of MOA proteins per indication is 1,142.2 (339); the maximal and minimal values are 15,281 (almost half of the total 32,584 screened proteins) for mast cell sarcoma and 0 for 82 diseases. The histogram of the number of MOAs is shown in Fig. 1b. 71.0% (40.6%) of indications have >100 (500) MOA proteins. These associations allowed us to expand the protein repertoire that might be associated with each disease and resemble the statistics from GWAS studies. Below, we describe the use of **LeMeDISCO** to predict disease comorbidities as well as prioritize these proteins.

### Shared MOA proteins explain disease comorbidity by way of disease–disease relationships

We next examine the overall characteristics of the predicted comorbidity network of 3608 diseases. Eighty-two diseases do not have MOA protein predictions and thus do not have predicted comorbidities. There are a total of 6,507,028 possible pairwise disease associations. Of these, there are 2,137,022 significant pairwise disease associations (*q* value <0.05) excluding the diagonals given by **LeMeDISCO**. Out of 3608, 3523 diseases have significant comorbidities. The density and frequency of the J-score for the significant non-redundant pairs is in Fig. 1c, and the density and frequency of the degree (number of edges) for each node (disease) is represented in Fig. 1d. Using a *q* value cutoff of 0.05, the average (median) number of comorbidities per disease is 608.3 (491). The largest (smallest) number of comorbidities is 2229 for Pneumonia aspiration and the smallest is 1 for these four diseases: glossopharyngeal neuralgia, median arcuate ligament syndrome, toxoplasmosis, hallucinogen dependence. The average closeness $\pm$ one standard deviation (defined as the reciprocal of the shortest distance to all other nodes: $\frac{{{{{{{\mathrm{Number}}}}}}\; {{{{{\mathrm{of}}}}}}\; {{{{{\mathrm{other}}}}}}\; {{{{{\mathrm{nodes}}}}}}}}{\sum {{{{{{\mathrm{shortest}}}}}}\; {{{{{\mathrm{distance}}}}}}\; {{{{{\mathrm{to}}}}}}\; {{{{{\mathrm{other}}}}}}\; {{{{{\mathrm{nodes}}}}}}}}$) of all nodes is 0.535 ± 0.084, indicating that the majority of disease pairs have the shortest distance around 1/0.535. The average betweenness is 1135 ± 1727, i.e., on average, 1135 pairs of diseases have their shortest distance passing through the given disease node. Thus, the disease network is very dense.

The cumulative distribution for the J-score and *q* values for all of the comorbidities and the top 100 are shown in Supplementary Figs. 5, 6, respectively. The summary statistics of the scores for these thresholds are shown in Supplementary Table 1. What is clear from these figures and Supplementary Table 1, particularly for the top 100 ranked comorbidities, is that the 99.6% top-ranked 100 comorbidities have a *q* value <0.005. In other words, while a *q* value threshold of 0.05 is used, in reality, the actual *q* values employed for subsequent analysis are far more significant.

Around 32.8% of the disease pairs have a *q* value <0.05. This result is consistent with the 37.1% recall of large-scale benchmarking (see Table 1). As shown in Fig. 1e, the giant component (GP) of the disease–disease network covers the entire network when the J-score is <0.1 and the *q* value <0.05, i.e., starting from any disease, one can walk to any other disease on the network. As the J-score cutoff increases, the number of diseases in the giant component decreases; however, the decrease is very slow. The rapid decrease only happens around a 0.45 J-score corresponding to an average *q* value of 1.63 × 10^−6^ ± 1.81 × 10^−4^. Thus, the disease network is not only dense, but it is also strongly (i.e., one has to apply a high J-score cutoff to break the network into small GP) and highly significantly (compared to default *q* value 0.05) connected. These issues will be explored in future work.

### LeMeDISCO identified MOA proteins

In addition to the comorbidity predictions, **LeMeDISCO** also identifies comorbidity enriched MOA proteins. The comorbidity enriched MOA proteins are hierarchically ranked by their CoMOAenrich score (defined in the Methods section). Comparing the top 100 comorbidity enriched MOA proteins (hierarchically ranked by the CoMOAenrich score) with the **MEDICASCY** top 100 MOA proteins (ranked by *q* value), 92.5% of the diseases have proteins with a significant overlap *p* value (<0.05). The cumulative distribution for the CoMOAenrich scores and *q* values for all the comorbidity enriched MOA proteins and the top 100 are shown in Supplementary Figs. 7, 8, respectively. The summary statistics of the scores for these thresholds are shown in Supplementary Table 1. For the comorbidity enriched MOA proteins ranked by their CoMOAenrich score, 65.9% have a *q* value <0.005. If one only assesses the top 100 comorbidity enriched MOA proteins, 67.1% have a *q* value <0.005, which are the proteins used for the global pathway analysis.

### Mapping of the LeMeDISCO MOA proteins to significant pathways

The cumulative distribution of the *q* values for the pathways and the top 100 pathways are shown in Supplementary Fig. 9 and the summary statistics are provided in Supplementary Table 1. As shown in Supplementary Fig. 9, 73.1% of the significant pathways (*q* value <0.05) have a *q* value <0.015. About 3453 or 95.7% of the 3608 diseases have significant pathways. We further note that there are some MOA proteins (e.g., AR, NR4A3, and PGR) and pathways (e.g., HSP90 chaperone cycle for steroid hormone receptors, SUMO E3 ligases SUMOylate target proteins, SUMOylation) that are present in approximately a third of the diseases in our library.

### Applications of LeMeDISCO

By way of illustration, we applied **LeMeDISCO** to two disparate diseases, coronary artery disease (CAD) and ovarian cancer (OC).

### Coronary artery disease (CAD)

CAD, a leading cause of death worldwide, is caused by narrowed or blocked arteries due to plaques composed of cholesterol or other fatty deposits lining the inner wall of the artery. These plaques result in decreased blood supply to the heart^20^. We find 2576 significant comorbid diseases (*q* value <0.05) and 785 (558) comorbidity enriched MOA proteins (genes) (score >0.01), meaning that at least one of the top 100 comorbid disease shares the protein as an MOA protein. This is the *p* value weighted comorbidity frequency normalized by the number of comorbid diseases used for calculating the frequency. See Methods for more details. Forty-nine significant pathways (*q* value <0.05) are associated with the top-ranked 100 comorbidity enriched proteins. The top 20 disease comorbidities, top 20 comorbidity enriched MOA proteins, and top 20 significant pathways are shown in Table 2. There are several significant cardiovascular-related comorbidities such as heart disease, cardiovascular system disease, myocardial infarction, and congestive heart failure. Asthma^21^, diabetes^22^, and obstructive lung disease^23^ are also in the top ten with known comorbidities to CAD. CAD is also known to be comorbid with liver disease^24^, kidney disease^25^, and hyperthyroidism^26^. Interestingly, allergic rhinitis is associated with decreased coronary heart disease^27^. In summary, 14 (70%) of the top 20 predicted comorbid diseases have literature evidence to support these predictions. To further show that these comorbidities with literature evidence cannot be generated randomly, we randomly selected 20 diseases from the 3608 diseases and did a literature search for their associations with CAD. We found nine diseases, far fewer than our list of 14 diseases (see Supplementary Table 2). A further random test selecting 20 from those after excluding LeMeDISCO predicted comorbid diseases to CAD, we find six diseases having literature evidence (see Supplementary Table 3).

**Table 2:** Top 20 comorbidities (excluding same disease pair, (i.e., CAD-CAD)), top 20 comorbidity enriched MOA proteins (with respect to original disease), and top 20 pathways associated with the prediction CAD results.

Comorbidities			MOA proteins		Pathways	
Disease	J-score	*q* value	Gene name	Score	Pathway	*q* value
Heart disease	0.47	<0.0001	COX7A2L	0.38	Class A/1 (Rhodopsin-like receptors)	3.7 × 10^−9^
Cardiovascular system disease	0.45	<0.0001	COX7A2	0.38	Olfactory signaling pathway	2.7 × 10^−8^
Obstructive lung disease	0.43	<0.0001	COX7A1	0.38	GPCR ligand binding	3.8 × 10^−8^
Asthma	0.44	<0.0001	NR4A3	0.36	The canonical retinoid cycle in rods (twilight vision)	4.5 × 10^−7^
Myocardial infarction	0.39	<0.0001	PGR	0.36	ADORA2B mediated anti-inflammatory cytokines production	1.4 × 10^−6^
Familial hyperlipidemia	0.33	<0.0001	LXN	0.35	Nuclear receptor transcription pathway	2.3 × 10^−6^
Diabetes mellitus	0.33	<0.0001	OSBPL8	0.35	Anti-inflammatory response favoring Leishmania parasite infection	7.6 × 10^−6^
Rhinitis	0.32	<0.0001	SLC8A3	0.35	Leishmania parasite growth and survival	7.6 × 10^−6^
Liver disease	0.31	<0.0001	KCNA10	0.35	Peptide ligand-binding receptors	3.3 × 10^−5^
Hyperthyroidism	0.31	<0.0001	NR3C2	0.35	SUMOylation of intracellular receptors	3.5 × 10^−5^
Chronic obstructive pulmonary disease	0.30	<0.0001	RARRES1	0.35	G alpha (i) signaling events	9.7 × 10^−5^
Lymphedema	0.29	<0.0001	GPRC5A	0.34	Visual phototransduction	1.3 × 10^−4^
Allergic asthma	0.29	<0.0001	ANXA1	0.34	Amine ligand-binding receptors	1.5 × 10^−4^
Intrinsic asthma	0.29	<0.0001	NR3C1	0.33	Leishmania infection	1.6 × 10^−4^
Pulmonary emphysema	0.29	<0.0001	ELOVL7	0.33	Integrin cell surface interactions	3.6 × 10^−4^
Syndrome	0.29	<0.0001	TSPAN13	0.33	Sodium/Calcium exchangers	9.6 × 10^−4^
Congestive heart failure	0.28	<0.0001	GRP	0.33	Retinoid cycle disease events	9.7 × 10^−4^
Kidney disease	0.28	<0.0001	ELOVL3	0.33	Diseases associated with visual transduction	9.7 × 10^−4^
pseudohypoparathyroidism	0.28	<0.0001	ELOVL1	0.32	Reduction of cytosolic Ca++ levels	9.7 × 10^−4^
Fatty liver disease	0.28	<0.0001	OSBPL5	0.32	Diseases of the neuronal system	9.7 × 10^−4^

Among the top, COX-related comorbidity enriched proteins were found. COX proteins are involved in the synthesis of prostanoids. Prostanoids are structurally like lipids and are involved in thrombosis and other undesirable cardiovascular events^28^. Several GPCR-related pathways (Class A/1 rhodopsin-like receptors, olfactory signaling pathway, GPCR ligand binding) are among the top five pathways predicted for CAD, consistent with the literature that GPCRs play a crucial role in heart function^29^.

The above results were obtained without any extrinsic knowledge of CAD. Next, we show how **LeMeDISCO** can be used to prioritize targets from other studies. A GWAS study identified 155 CAD-associated genes^30^. While they are associated with CAD, to find out which ones to target is a non-trivial task. Here, we applied **LeMeDISCO** to prioritize them by examining their frequencies of presence in other diseases. There were 26 comorbidity enriched MOA proteins (score >0.01) and 40 pathways (*p* value <0.05, but *q* value <0.20) found from global pathway analysis of the 26 comorbidity enriched MOA proteins. The top disease comorbidities, top 20 comorbidity enriched MOA proteins, and top 20 pathways are shown in Table 3. There were only three significant predicted comorbidities (*q* value <0.05) by **LeMeDISCO**. The top two comorbidities are renal artery disease and anuria, both are associated with dysfunction of the kidneys and are related to CAD^25,31^. Anuria is attributed to failure of the kidneys to produce urine, and renal artery disease occurs when the arteries that supply blood and oxygen to the kidneys narrows. A study found an increase in renal artery stenosis in patients with CAD^31^. The last comorbid disease is anterior uveitis. Studies showed that anterior uveitis is associated with Kawasaki disease that can lead to heart complication^32^. Thus, all three have literature evidence.

**Table 3:** Up to top 20 comorbidities, top 20 comorbidity enriched MOA proteins (with respect to input), and top 20 pathways (ranked by *p* value since *q* values are the same) associated with the prediction CAD GWAS-driven LeMeDISCO results using the gene set from ref. ^30^.

Comorbidities			MOA proteins		Pathways		
Disease	J-score	*q* value	Gene name	Score	Pathway	*q* value	*p* value
Renal artery disease	0.028	5.8 × 10^−4^	PEX10	0.24	RAB geranylgeranylation	0.16	4.6 × 10^−3^
Anuria	0.022	5.3 × 10^−3^	BEND6	0.23	Platelet activation, signaling and aggregation	0.16	7.7 × 10^−3^
Anterior uveitis	0.015	0.022	NEURL1	0.22	MET activates RAP1 and RAC1	0.16	0.017
			CCM2	0.20	RHO GTPases activate KTN1	0.16	0.017
			FGD6	0.20	Response to elevated platelet cytosolic Ca2+	0.16	0.019
			CENPW	0.20	Killing mechanisms	0.16	0.019
			PCID2	0.20	WNT5:FZD7-mediated leishmania damping	0.16	0.019
			RPL17	0.19	Diseases of signal transduction by growth factor receptors and second messengers	0.16	0.022
			MANEAL	0.18	PTK6 Regulates RHO GTPases, RAS GTPase, and MAP kinases	0.16	0.022
			HHAT	0.17	TFAP2 (AP-2) family regulates the transcription of growth factors and their receptors	0.16	0.024
			PHYHIP	0.16	Purine catabolism	0.16	0.030
			IYD	0.16	RHO GTPases activate CIT	0.16	0.031
			VEGFA	0.16	Signal transduction by L1	0.16	0.033
			HNRNPD	0.14	VEGFR2 mediated cell proliferation	0.16	0.033
			AGT	0.13	RHO GTPases Activate NADPH Oxidases	0.16	0.037
			PLEKHA1	0.12	RHO GTPases activate PAKs	0.16	0.037
			SERPINA1	0.11	TRAF6 mediated NF-kB activation	0.16	0.037
			NUDT5	0.04	Neutrophil degranulation	0.16	0.038
			RAB23	0.04	NOTCH3 Activation and Transmission of Signal to the Nucleus	0.16	0.039
			NKIRAS2	0.04	Signaling by NTRK2 (TRKB)	0.16	0.039

While the top genes are associated with CAD according to the GWAS study of ref. ^30^, we predicted that they are also associated with the corresponding comorbid diseases—renal artery disease, anuria, and anterior uveitis. For example, VEGFA is predicted to be associated with all three comorbid diseases. It was found that in progressive kidney disease, the VEGFA expression level is attenuated^33^; in contrast, in uveitis disease, it is increased^34^. Other top genes are predicted to be associated only with anterior uveitis. Among them, SERPINA1 is a potential causal gene of uveitis^35^, RAB23 is associated with uveitis in sarcoidosis^36^, and HHAT has evidence of association with uveitis^37^.

None of the 40 pathways obtained using the top 26 genes overlaps with the six pathways obtained using the original 155 genes with the same cutoff. The predicted top pathway RAB geranylgeranylation through RAB23/RAB5C genes is part of the signaling network of statin-induced effects of improving cardiac health in Drosophila^38^.

### Ovarian cancer (OC)

**LeMeDISCO** predicts 1,092 significant comorbidities to OC (*q* value <0.05), with 282 (171) comorbidity enriched MOA proteins (genes) (score >0.01). There were 159 significant pathways (*q* value <0.05) from the top 100 comorbidity enriched MOA proteins. The top 20 disease comorbidities, top 20 comorbidity enriched MOA proteins, and all significant pathways are shown in Table 4. It is not surprising that all of the top comorbidities are cancers. The top first comorbid disease is testicular cancer. Although OC and testicular cancer cannot occur in one individual, they are hereditarily associated^39^. Fallopian tube cancer is considered similar to OC. It was reported that squamous cell carcinoma occurred in the ovary^40^. Nodular prostate, (the male version of OC), cervical cancer^41^, and inflammatory breast carcinoma^42^ are all reproduction-related cancers like OC. OC from lung cancer metastasis occurs in 2–4% of OC patients^43^. Bile duct cancer is a very rare site of OC metastases^44^. Peritoneal cancer behaves similarly to OC. Gland cancer is linked to BRCA-positive families, and BRCA is a risk gene for ovarian cancer^45^. Neurofibroma is reported to mimic ovarian tumors^46^. Renal cell carcinoma is metastatic to ovarian and fallopian tube cancers^47^. In total, 14 of the top 20 (70%) comorbidities have literature evidence. Similar to CAD, we did a literature search of 20 randomly selected diseases for their associations with OC. We found only 6 cases; far fewer than our 14 (see Supplementary Table 2). In a further random test selecting 20 from those after excluding **LeMeDISCO** predicted comorbid diseases to OC, we find four diseases have literature evidence (see Supplementary Table 3).

**Table 4:** Top 20 comorbidities (excluding same disease pair, (i.e., OC-OC)), top 20 comorbidity enriched MOA proteins (with respect to original disease), and top 20 pathways associated with the prediction OC results.

Comorbidities			MOA proteins		Pathways	
Disease	J-score	*q* value	Gene name	Score	Pathway	*q* value
testicular cancer	0.42	<0.0001	TEK	0.5	MAPK1/MAPK3 signaling	7.10 × 10^−15^
fallopian tube cancer	0.41	<0.0001	TYRO3	0.49	EPH-Ephrin signaling	8.59 × 10^−15^
squamous cell carcinoma	0.40	<0.0001	RYK	0.49	RAF/MAP kinase cascade	2.37 × 10^−14^
tongue squamous cell carcinoma	0.39	<0.0001	MERTK	0.49	MAPK family signaling cascades	2.69 × 10^−14^
nodular prostate	0.36	<0.0001	AXL	0.49	FLT3 Signaling	3.83 × 10^−14^
cervical cancer	0.36	<0.0001	LTK	0.48	EPH-ephrin-mediated repulsion of cells	3.96 × 10^−14^
myeloproliferative neoplasm	0.32	<0.0001	EGFR	0.47	PI5P, PP2A, and IER3 Regulate PI3K/AKT Signaling	4.07 × 10^−13^
inflammatory breast carcinoma	0.31	<0.0001	KIT	0.47	Negative regulation of the PI3K/AKT network	9.15 × 10^−13^
urinary bladder cancer	0.30	<0.0001	KDR	0.47	Constitutive Signaling by Aberrant PI3K in Cancer	3.79 × 10^−12^
lung cancer	0.30	<0.0001	FLT3	0.47	PI3K/AKT Signaling in Cancer	1.3 × 10^−10^
bile duct cancer	0.29	<0.0001	FLT1	0.47	EPHA-mediated growth cone collapse	5.51 × 10^−10^
parotid gland cancer	0.29	<0.0001	ROR2	0.47	Diseases of signal transduction by growth factor receptors and second messengers	3.45 × 10^−9^
neurofibroma	0.29	<0.0001	RET	0.47	PIP3 activates AKT signaling	8.38 × 10^−8^
peritoneum cancer	0.28	<0.0001	PTK2B	0.47	EPHB-mediated forward signaling	2.83 × 10^−7^
gallbladder cancer	0.28	<0.0001	PTK2	0.47	Intracellular signaling by second messengers	4.76 × 10^−7^
Barrett’s esophagus	0.28	<0.0001	NTRK3	0.47	Toll-like receptor 4 (TLR4) cascade	4.87 × 10^−6^
tongue cancer	0.27	<0.0001	NTRK2	0.47	Toll-like receptor cascades	2.16 × 10^−5^
larynx cancer	0.27	<0.0001	NTRK1	0.47	ERBB2 activates PTK6 signaling	2.23 × 10^−5^
kidney cancer	0.27	<0.0001	MUSK	0.47	ERBB2 regulates cell motility	3.98 × 10^−5^
lung benign neoplasm	0.27	<0.0001	LMTK3	0.47	PI3K events in ERBB2 signaling	5.02 × 10^−5^

Eleven of the top 20 enriched MOA proteins are kinases that are cancer-related. The topmost comorbidity enriched MOA protein is TEK, angiopoietin-1 receptor; angiopoietins are found to promote ovarian cancer progression^48^. Interestingly, TYRO3 is related to drug resistance in OC^49^. The top predicted pathway by enriched MOAs is MAPK1/MAPK3 signaling that mediates the expression of ERBB2 silencing, OC cell migration, and invasion^50^. There are also enriched pathways associated with ephrin ligands. Aggressive forms of ovarian cancer have been previously shown to involve upregulated forms of ephrin, such as ephrinA5^51^. There are 14 ephrin-related comorbidity enriched MOA proteins found (all score >0.37).

We next examined a set of 11 genes associated with OC risk from a study that assessed the multiple-gene germline sequences in 95,561 women with OC using **LeMeDISCO**^52^. The results for the top 20 comorbidities, seven MOA proteins (score >0.01), and their associated pathways are shown in Table 5. There were 125 significant comorbidities (*q* value <0.05) predicted and 33 significant pathways (*q* value <0.05) associated with these seven proteins. The top comorbidity associated with OC was angiosarcoma, a rare cancer of the inner blood and lymph vessels and in very rare cases, it occurs in the ovaries^53^. Patients with epithelial ovarian cancers show an increased risk of skin cancer^54^. OC is also considered to have genetic risk factors^55^. Myxoid leiomyosarcoma is a very rare tumor with similarity to ovarian cancer^56^, and leiomyosarcoma was reported in the ovaries^57^. A study found a relationship between hemoglobin levels and interleukin-6 levels in individuals with untreated epithelial ovarian cancer, indicating an inflammatory role in cancer-associated anemia^58^. Medulloblastoma can arise from ovarian tumors in pregnancy^59^. Uveal cancer is associated with breast cancer and OC^60^. OC is part of urinary system neoplasm. In total, 15 (75%) of top 20 comorbidities have literature evidence.

**Table 5:** Top 20 comorbidities, seven comorbidity enriched MOA proteins (with respect to input), and top 20 pathways associated with the prediction OC GWAS-driven results using the gene set from ref. ^52^.

Comorbidities			MOA proteins		Pathways	
Disease	J-score	*q* value	Gene name	Score	Pathway	*q* value
angiosarcoma	0.0047	0.012	RAD51C	0.41	DNA repair	3.80 × 10^−8^
skin cancer	0.0036	0.012	RAD51D	0.39	Diseases of DNA repair	6.48 × 10^−8^
skin benign neoplasm	0.0036	0.012	MSH6	0.39	Mismatch repair	1.23 × 10^−7^
ovarian carcinoma	0.0036	0.024	MSH2	0.25	Mismatch repair (MMR) directed by MSH2:MSH6 (MutSalpha)	1.23 × 10^−7^
biliary tract disease	0.0035	0.028	MLH1	0.12	Resolution of D-loop structures through synthesis-dependent strand annealing (SDSA)	5.59 × 10^−7^
genetic disease	0.0033	0.012	BRIP1	0.065	Transcriptional regulation by TP53	8.02 × 10^−7^
myxoid leiomyosarcoma	0.0032	0.017	STK11	0.050	Resolution of D-loop structures	8.02 × 10^−7^
epithelioid leiomyosarcoma	0.0032	0.017			Resolution of D-loop structures through holliday junction intermediates	8.02 × 10^−7^
leiomyosarcoma	0.0032	0.017			Presynaptic phase of homologous DNA pairing and strand exchange	1.09 × 10^−6^
mesenchymoma	0.0031	0.019			Homologous DNA pairing and strand exchange	1.23 × 10^−6^
hematopoietic system disease	0.0031	0.019			TP53 regulates the transcription of DNA repair genes	4.22 × 10^−6^
lymphatic system disease	0.0031	0.039			HDR through homologous recombination (HRR)	4.24 × 10^−6^
childhood medulloblastoma	0.0031	0.012			Mismatch repair (MMR) directed by MSH2:MSH3 (MutSbeta)	1.61 × 10^−5^
adult medulloblastoma	0.0031	0.012			HDR through homologous recombination (HRR) or single-strand annealing (SSA)	2.79 × 10^−5^
medullomyoblastoma	0.0031	0.012			Homology directed repair	2.98 × 10^−5^
chondrosarcoma	0.0031	0.019			DNA double-strand break repair	4.83 × 10^−5^
pancreas disease	0.0031	0.041			Meiotic recombination	4.85 × 10^−4^
metachromatic leukodystrophy	0.0030	0.021			Regulation of TP53 activity through phosphorylation	5.23 × 10^−4^
uveal cancer	0.0030	0.021			Meiosis	8.11 × 10^−4^
urinary system benign neoplasm	0.0029	0.024			Reproduction	1.12 × 10^−3^

The top two comorbidity enriched MOA proteins are RAD51C, RAD51D and belong to 16 of the top 20 pathways. These involve such processes as DNA repair, transcriptional regulation by TP53, DNA double-strand break repair, and reproduction (see Table 5). The top two and the third-ranked MSH6 proteins are shared by all top 100 comorbidities. For example, RAD51C is associated with Fanconi anemia (ranked 63th)^61^; RAD51D is associated with leiomyosarcoma^62^, and MSH6 is a risk gene for pancreatic adenocarcinoma (26th)^63^.

### LeMeDISCO web server

The **LeMeDISCO** web service allows researchers to query our library of 3608 diseases or input a set of pathogenic human genes/proteins and compute their predicted comorbidities, prioritized MOA proteins, and pathways associated. The web service is freely available for academic users at http://sites.gatech.edu/cssb/LeMeDISCO. The programs and input data for reproducing disease–protein, disease–disease relationships, and all **LeMeDISCO** results as well as benchmark results are available at https://github.com/hzhou3ga/lemedisco.

## Discussion

**LeMeDISCO** is a systematic approach for studying and analyzing possible features underlying the common proteins driving comorbid diseases. The resulting predicted driver proteins and pathways for each disease or input gene set can allow researchers to generate new diagnostic and treatment options and hypotheses. Interestingly, there were some MOA proteins and pathways present across approximately a third of the diseases, implying common disease drivers. The implications of this observation and its relationship to disease origins will be pursued in future work. We do note that the current comorbid disease analysis strongly suggests that the “one target-one disease-one molecule” approach often used in developing disease therapeutics^31^ is likely too simplistic.

To fully understand the complexities of a disease, one must trace the origin of its pathogenesis, which may be due to a genetic or somatic variant that is somehow related to the disease. However, such variants may also be associated with a disease not previously known to be associated with that disease. Such interrelations can be further investigated by identifying high confidence comorbidity predictions from **LeMeDISCO**, regardless of whether or not their comorbidity was previously known in the literature. For example, analysis of the comorbid diseases associated with CAD and OC have not only recapitulated known disease comorbidities but have also provided novel insights. The results for CAD yielded high confidence associations between liver diseases and forms of asthma, which can be further investigated through the comorbidity enriched MOA proteins and pathways. Furthermore, the results for OC revealed more high confidence associations to other forms of cancer such as squamous cell carcinoma and lung cancer.

**LeMeDISCO** not only has applications to the study of the underlying etiology behind a disease but may also be used during the early stages of drug discovery to identify efficacious drugs. Rather than starting with a small molecule or protein target of choice, **LeMeDISCO** allows one to begin at the level of disease biology, often termed phenotypic drug discovery. In future work, we shall demonstrate the utility of **LeMeDISCO** in identifying efficacious drugs to treat a given disease. Overall, the results of the current analysis and preliminary applications to drug discovery suggest that **LeMeDISCO** provides a set of tools for elucidating disease etiology and interrelationships and that a more systems-wide, comprehensive approach to both personalized medicine and drug discovery is required.

We note that some of the predicted MOA proteins are present in around 1/3 of diseases. The top five (AR, NR4A3, PGR, NR3C2, and NR3C1) proteins all belong to the nuclear receptor family and regulate other genes. All have DNA binding sites, especially two zinc finger domains^64^. The regulatory functions and ubiquity of well-studied zinc fingers in these proteins may explain their frequent presentation as disease MOA proteins^65^. Even though in our predicted drug targets of the probe drugs, these proteins are not the most frequent ones (e.g., AR is ranked 1135th of 16,762), their disease associations were enriched by MEDICASCY predicted disease–drug relationships.

With the above possible applications, there is also the limitation of the current approach of using FDA-approved DrugBank drugs as probe drugs to tease out the MOA proteins of diseases, i.e., some possible MOA proteins of a given disease might not be the targets of the probe drugs and others might be incorrectly assigned. This will be addressed in future work that includes more diverse small molecule drug libraries and improved virtual ligand screening algorithms to map the drugs to their respective protein targets^66^. As the probe drug target space is expanded, additional MOA proteins will be discovered. Concomitantly, as the virtual ligand screening algorithms that assign small molecules to their predicted protein targets improve, false positives will be eliminated, and additional true positive proteins might be added. These will result in more accurate MOA protein predictions.

Similar to previous work^1,6–8^, our predicted disease–disease relationships were benchmarked using large-scale clinical data and have only small-scale validation by literature searches. One single relationship requires at least one published work to validate. Large-scale automatic text mining is a feasible way to scale up the validation and build a more confident subset of our predictions^67^. This is the subject of ongoing studies.

## Methods

### Overview of LeMeDISCO

**Fig. 2:**
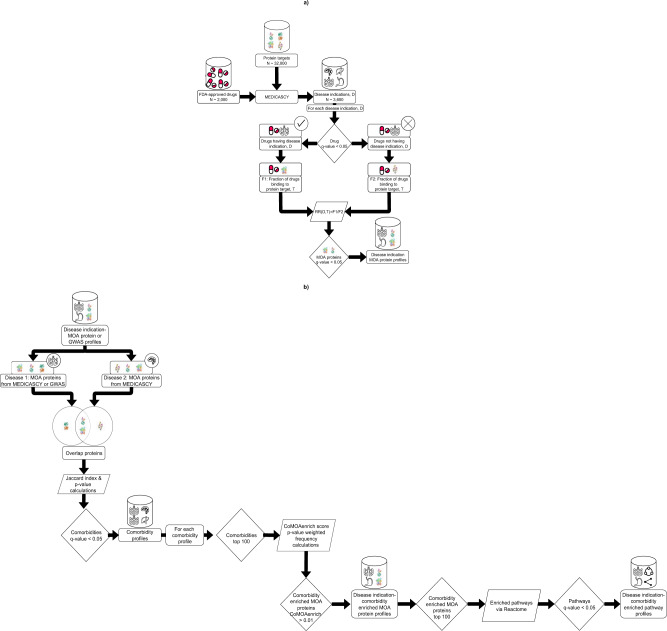
Schematic representation of **LeMeDISCO**. **a** The method for determining the MOA proteins associated with a disease indication via **MEDICASCY**, and **b** The method for determining the comorbidities associated with a given disease and its molecular mechanisms via LeMeDISCO.

A flowchart of **LeMeDISCO** is shown in Fig. 2. **LeMeDISCO** employs **MEDICASCY**^12^ to predict possible disease MOA proteins. Here, **MEDICASCY** is applied in prediction mode (i.e., any training drugs having a Tanimoto-Coefficient = 1 to a given input drug is excluded from training) to avoid a strong bias toward drugs in the training set on a set of 2095 FDA-approved drugs^68^. For each of the 3608 indications, we rank the 2095 probe drugs according to their *Z*-scores, *Z*_*d*_, defined using the raw score computed by **MEDICASCY** from:

1 $${Z}_{d}=\left(\frac{{{{{{{\mathrm{raw}}}}}}}\,{{{{{{\mathrm{score}}}}}}}{{\mbox{--}}}{{{{{{\mathrm{average}}}}}}}\,{{{{{{\mathrm{raw}}}}}}\,{{{{{\mathrm{score}}}}}}}\,{{{{{{\mathrm{of}}}}}}}\,2095\,{{{{{{\mathrm{drugs}}}}}}}}{{{{{{{\mathrm{standard}}}}}}}\,{{{{{{\mathrm{deviation}}}}}}}\,{{{{{{\mathrm{of}}}}}}}\,2095\,{{{{{{\mathrm{raw}}}}}}}\,{{{{{{\mathrm{scores}}}}}}}}\right)$$

To predict a drug as having the given indication, we applied a *Z*_*d*_ cutoff of 1.65, that approximately corresponds to a *p* value of 0.05 for the upper-tailed null hypotheses of random variable *Z*_*d*_. Thus, for each indication D, the 2095 probe drugs are separated into two groups: *N*_1_ are predicted to have indication D (*Z*_*d*_ ≥ 1.65) and *N*_2_ (=2095 − *N*_1_) are not predicted to have indication *D* (*Z*_*d*_ < 1.65). This is a very loose prediction of a drug’s indication with the advantage that it always predicts some drugs having the indication with its expected statistical confidence. Then, for a given indication D and each protein target, T, in the human proteome of our modeled 32,584 proteins, there are a subset of the drugs (or perhaps none) predicted by **FINDSITE**^**comb2.0**^ ^69^ to bind T. The relative risk RR(D, T) of the given target T with respect to indication D as:

2a $${{{{{\rm{RR}}}}}}({{{{{\rm{D}}}}}},{{{{{\rm{T}}}}}})=\frac{{N}_{1}^{T}/{N}_{1}}{{N}_{2}^{T}/{N}_{2}}$$

where ${N}_{1}^{T}$ and ${N}_{2}^{T}$ are the numbers of drugs binding to T with and without indication D, respectively. The numerator is the estimation of the probability of drugs having the predicted indication D (*Z*_*d*_ ≥ 1.65) that bind to protein T (F1 = ${N}_{1}^{T}/{N}_{1}$). The denominator is the probability of finding drugs that do not have the predicted indication D but which bind to protein T (F2 = ${N}_{2}^{T}/{N}_{2}$). This latter probability serves as the background probability that an arbitrary drug will bind to T. When no drug is predicted to bind to protein T, RR(D, T) is set to zero. RR(D, T) = F1/F2 >1 means that a drug having indication D is more likely to bind to T than arbitrary drugs not having the predicted indication D will bind to T.

We then compute the statistical significance of RR(D, T) by calculating a *p* value using Fisher’s exact test^70,71^ on the following contingency table:

2b $$\left(\begin{array}{cc}{N}_{1}^{T} & {N}_{1}-{N}_{1}^{T}\\ {N}_{2}^{T} & {N}_{2}-{N}_{2}^{T}\end{array}\right)$$

We define a protein target T as predicted to be a possible MOA target for indication D if its *p* value <0.05 because it is more likely to be targeted by efficacious drugs than arbitrary drugs. Thus, for each of the 3608 indications, there is a list of predicted possible MOA proteins.

To reduce false positive MOAs, we utilized the human protein atlas database (https://www.proteinatlas.org/about/download, *normal_tissue.tsv*) of expression profiles for proteins in normal human tissues based on immunohistochemistry using tissue micro arrays^72^ to filter those proteins that are “not detected” and not “uncertain” in all tested tissues related to an indication. To determine the tissues related to an indication, tissues are mapped to their ICD-10 main codes and indications having the same main codes are related to the tissue.

Using the input of two sets of putative MOA proteins having a *p* value of <0.05 calculated by Fisher’s exact test^70^, we calculate their Jaccard index^17^ J(D_1_,D_2_) (J-score) defined in Eq. 3a as

3a $${{{{{\rm{J}}}}}}{\mbox{-}}{{{{{\rm{score}}}}}}={N}_{s}/({{{{{\rm{N}}}}}}{{{{{\rm{D}}}}}}1+{{{{{\rm{N}}}}}}{{{{{\rm{D}}}}}}2-{N}_{s})$$

We then calculate the *p* value for significance by Fisher’s exact test for the contingency table^70^ that gives the probability of having overlap ≥*N*_*s*_ by randomly selecting ${N}_{D2}$ out of ${N}_{t}$ proteins^70,73^:

3b $$\left(\begin{array}{cc}{N}_{s} & {N}_{D2}-{N}_{s}\\ {N}_{D1} & {N}_{t}-{N}_{D1}\end{array}\right)$$

${N}_{D1}$, ${N}_{D2}$ are the numbers of MOA proteins/genes of disease D_1_ and D_2_; *N*_*s*_ is the number of overlapped MOA proteins between D_1_, D_2_, and ${N}_{t}$ is the total number of human proteins. The Jaccard index J-score is a statistical measure of the similarity between MOA proteins of D_1_ and D_2,_ and its value ranges between 0 and 1. Since the null hypothesis of ${N}_{s}$ corresponds to a hypergeometric distribution, the *p* value of observing the number of overlapped MOA proteins between D_1_, D_2_ $\ge$ ${N}_{s}$ can be calculated using Fisher’s exact test on the table in Eq. 3b^71^. We will use the J-score for predicting comorbidity and compare it with the observed comorbidity. We note that the J-score is determined by the number of overlapped MOAs, which means that the comorbidity defined by the J-score are not limited by diseases occurring in one individual but rather considers the effect of the malfunctioning proteins in the human population. This is especially true for sex-specific diseases such as ovarian cancer and prostate cancer that may have overlapping MOA proteins; this may result in significant comorbidity between them. In other words, the two diseases may share common driver proteins, although ovarian and prostate cancer could occur unless the individual has both an ovary and a prostate, which is highly unlikely. Similarly, it can predict the comorbidity of rare and common diseases; again, whether this would occur would depend on the presence in a given person of the appropriate set of malfunctioning genes. Therefore, though many of the LeMeDISCO comorbidity predictions are seen in one individual, others may not be. Thus, LeMeDISCO comorbidity predictions are a population-based approach.

To better control the false discovery rate (FDR) due to background noise from statistic errors, we performed the multiple testing correction to the *p* values for disease–protein and disease–disease associations calculated by Fisher’s exact test by computing the *q* value using the method described in ref. ^74^.

In large-scale disease–disease comorbidity calculations, we use the MOAs predicted by **MEDICASCY**^12^. In addition, MOA targets between disease pairs can also be derived from experimental data; examples include differential gene expression (GE), Mendelian or somatic mutation profiles comparing disease vs. control normal samples, better vs. worse prognosis samples, or drug-treated vs. control untreated samples^75^.

### Benchmarking of LeMeDISCO

We validated **LeMeDISCO**’s J-score by correlating it with the observed comorbidity as quantified by (a) the logarithm of relative risk log(RR) score and (b) the φ**-**score (Pearson’s correlation for binary variables)^1^. The relative risk (RR) is the probability that two diseases co-occur in a single individual relative to random. Since RR scales exponentially with respect to the strength of two interacting diseases, we use log(RR) for correlation analysis. The log(RR) and φ**-**score are computed from US Medicare insurance claim data using^1^:

4a $${{\log }}({{{{{\rm{RR}}}}}})\,=\,{{\log }}\left(\frac{{n}_{{AB}}/{n}_{{tot}}} {({n}_{A}/{n}_{{tot}}){({n}_{B}/{n}_{{tot}})}}\right)$$

4b $${{\varphi }}{\mbox{-}}{{{{{\rm{score}}}}}}=({n}_{{AB}}* {n}_{{tot}}-{n}_{A}* {n}_{B})/\sqrt{{n}_{A}* {n}_{B}* \left({n}_{{tot}}-{n}_{A}\right)* \left({n}_{{tot}}-{n}_{B}\right)}$$

where *n*_*tot*_ = total number of patients; *n*_*A*_, *n*_*B*_ = number of patients diagnosed with diseases A and B, and *n*_*AB*_ = number of patients diagnosed with both diseases A and B.

### Permutation tests

Two permutation tests were performed: (a) Permute drug–protein relationships: Randomly permute the predicted drug–protein relations (i.e., randomly replace a drug’s protein targets with another drug’s protein targets predicted by **FINDSITE**^**comb2.0**^). This acts to transfer the protein targets of a drug (possibly incorrectly) to another drug. This test evaluates the performance of **LeMeDISCO** if we have the correct drug–disease relations (predicted by **MEDICASCY**) but the incorrect drug–protein relations. To ensure the correct drug–disease relations after permuting the drug–protein relations, **MEDICASCY** was applied to the permuted drug–protein relations since **MEDICASCY** depends on the drug’s protein targets; (b) Permute drug–disease relationships: Randomly permute the predicted drug–disease relations (by randomly replacing a drug’s predicted indications with another drug’s indications). This test evaluates how **LeMeDISCO** will perform if the drug–protein relations are correct (predicted by **FINDSITE**^**comb2.0**^), but the drug–disease relations are randomly permuted. In both cases, disease MOAs are derived using the permuted relationships and 100 runs for each test with different random seeds were performed. A *p* value is calculated from *z*-score = (LeMeDISCO value-average)/standard deviation to characterize the significance of the difference between **LeMeDISCO** and the permutation tests.

### Identification of key MOA proteins and associated pathways for disease comorbidity

After determining the significant comorbidities for each disease, the *p* value weighted frequency of shared MOA proteins across the top 100 predicted comorbidities are calculated. We define a *p* value weighted frequency of an input MOA as follows (i.e., CoMOAenrich score): If MOA protein T is shared by a comorbid indication D and the *p* value of T associated with D is *P*, then the weight defined by the min(1.0,−αlog*P*) is counted as T’s frequency. In practice, we used 10 cancer cell line data^76^ to optimize the coefficient α to 0.025. We further computed a *p* value via ${e}^{-\frac{{{{{{{\mathrm{COMOAenrich}}}}}}\; {{{{{\mathrm{score}}}}}}}}{\alpha }}$ where α = 0.025, as previously mentioned. These MOA proteins expand the number of possible molecular players driving disease pathogenesis. An empirically derived CoMOAenrich score (normalized by the number of comorbid indications that is 100) threshold of 0.01 was used, which is equivalent to 1% of the comorbid indications having the MOA proteins with a significant *p* value (<4.2 × 10^−18^). Then, up to the top 100 comorbidity enriched MOA proteins for each disease were used in global pathway analysis via Reactome^13^. The pathways with a *p* value <0.05 were extracted. The frequency of pathways across diseases was assessed to identify common pathways of disease.

### LeMeDISCO usage

As shown in Fig. 2, **LeMeDISCO** can be used in two different ways: (1) **MEDICASCY**-driven **LeMeDISCO**: The comorbidities for any of the 3608 diseases from the **MEDICASCY**-provided MOA proteins are predicted (Fig. 2a). (2) Pathogenic gene set driven **LeMeDISCO**: Input your own pathogenic gene set derived from differential gene expression, GWAS, exome analysis, or other experimental/clinical techniques (shown in Fig. 2b). The **LeMeDISCO** web service allows users to query the **LeMeDISCO** database as well as input their own set of pathogenic genes to assess the associated comorbidities, MOA proteins, and pathways.

### Reporting summary

Further information on research design is available in the Nature Research Reporting Summary linked to this article.

## Supplementary information

Supplementary Information

Description of Additional Supplementary Files

Supplementary Data 1

Reporting Summary

## Data Availability

The input data for reproducing disease–protein, disease–disease relationships, and all **LeMeDISCO** results as well as benchmark results are available at https://github.com/hzhou3ga/lemedisco. The web service is freely available for academic users at http://sites.gatech.edu/cssb/LeMeDISCO. The underline data for Fig. 1 is in file supplementary data 1.

## Code availability

The programs for reproducing disease–protein and disease–disease relationships are available at https://github.com/hzhou3ga/lemedisco.
