# Supplementary material for: LeMeDISCO is a computational method for large-scale prediction & molecular interpretation of disease comorbidity
Source: Commun Biol. 2022 Aug 25;5:870. doi: 10.1038/s42003-022-03816-9 (PMC9411158; doi:10.1038/s42003-022-03816-9)
Supplement: Supplementary file 1 — Supplementary Information [file 42003_2022_3816_MOESM1_ESM.pdf]

Supplementary Materials

LeMeDISCO is a computational method for large-scale prediction & molecular interpretation of disease comorbidity

Courtney Astore, Hongyi Zhou , Bartosz Ilkowski, Jessica Forness and Jeffrey Skolnick

Supplementary Figures

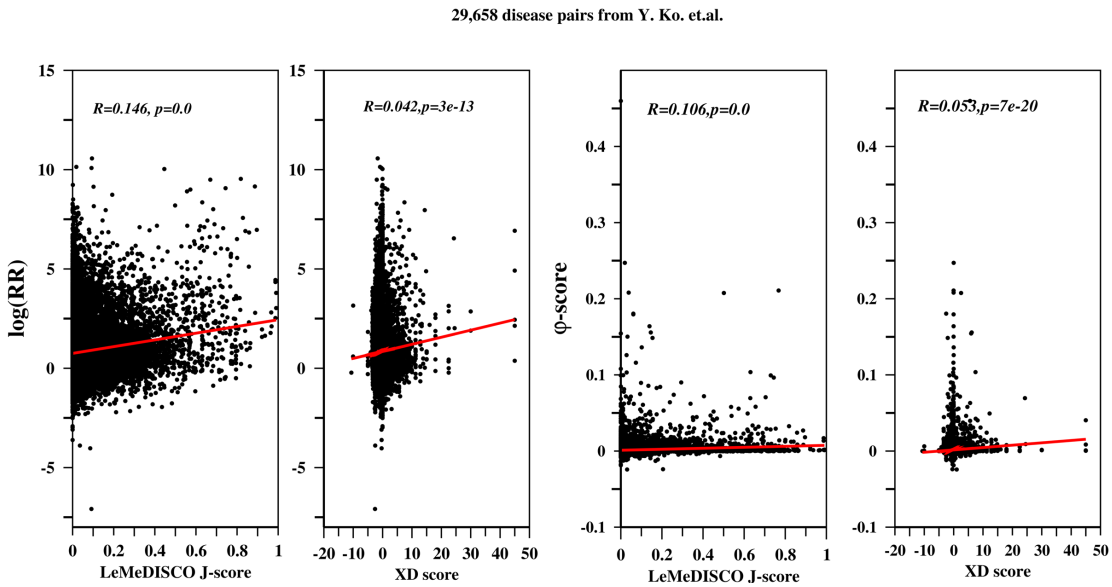

Supplementary Figure 1. Correlation between the XD score[8] and J-score to the log(RR) score and φ-score.

943 disease pairs from J. Menche et.al.

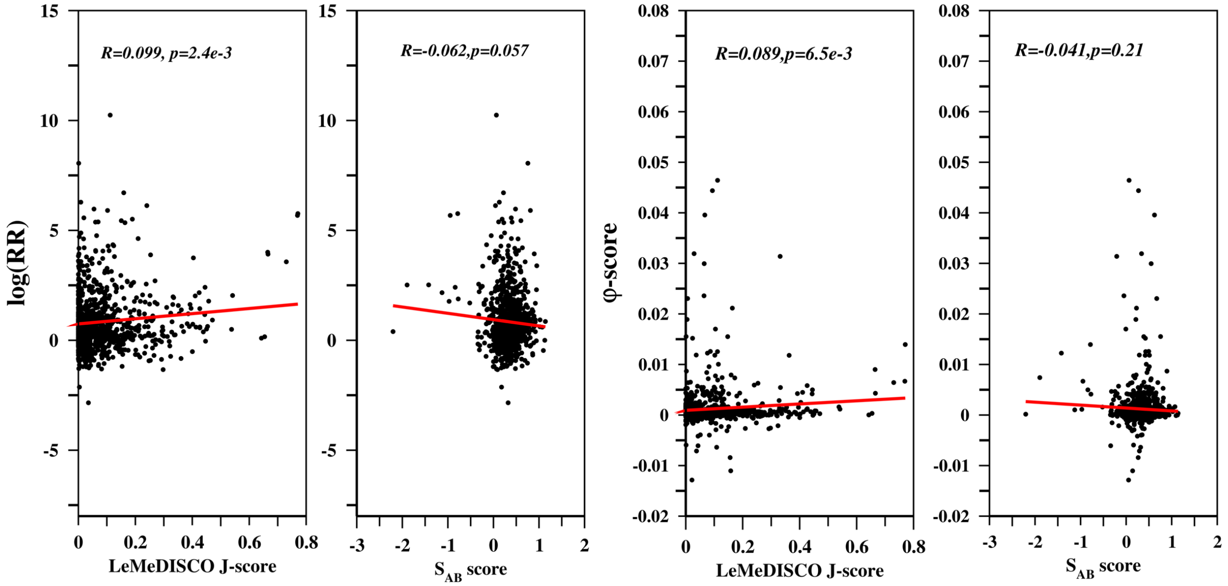

Supplementary Figure 2. The correlation between the S_{AB} score [7] and J-score to the log(RR) score and φ-score.

2,621 disease pairs from X. Zhou et.al.

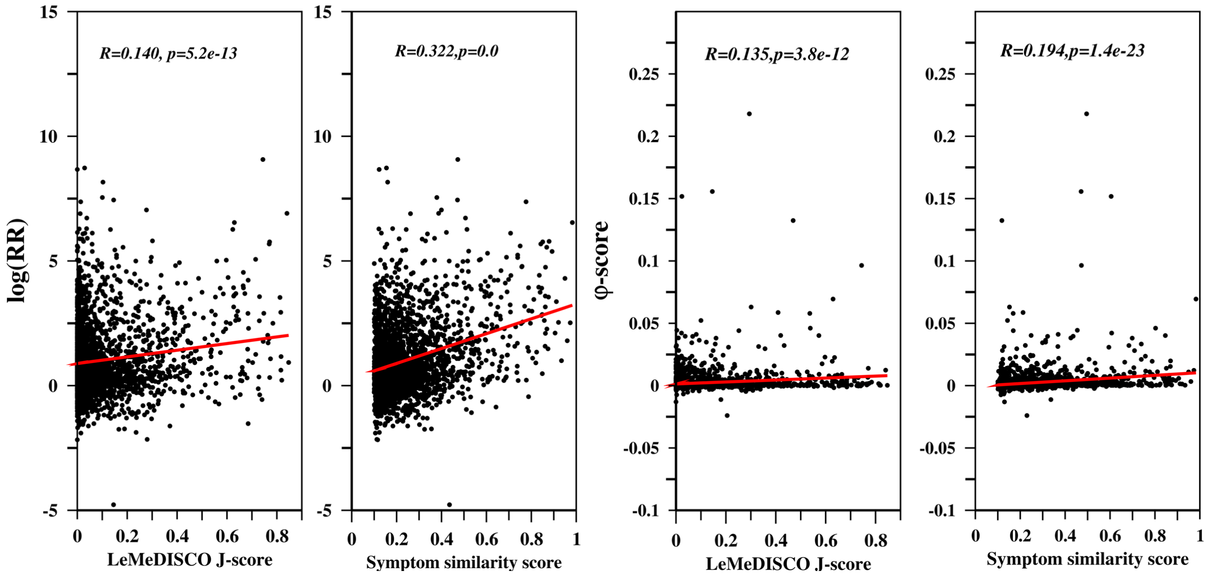

Supplementary Figure 3. Correlation between the Symptom Similarity Score [6] and J-score to the log(RR) score and φ-score.

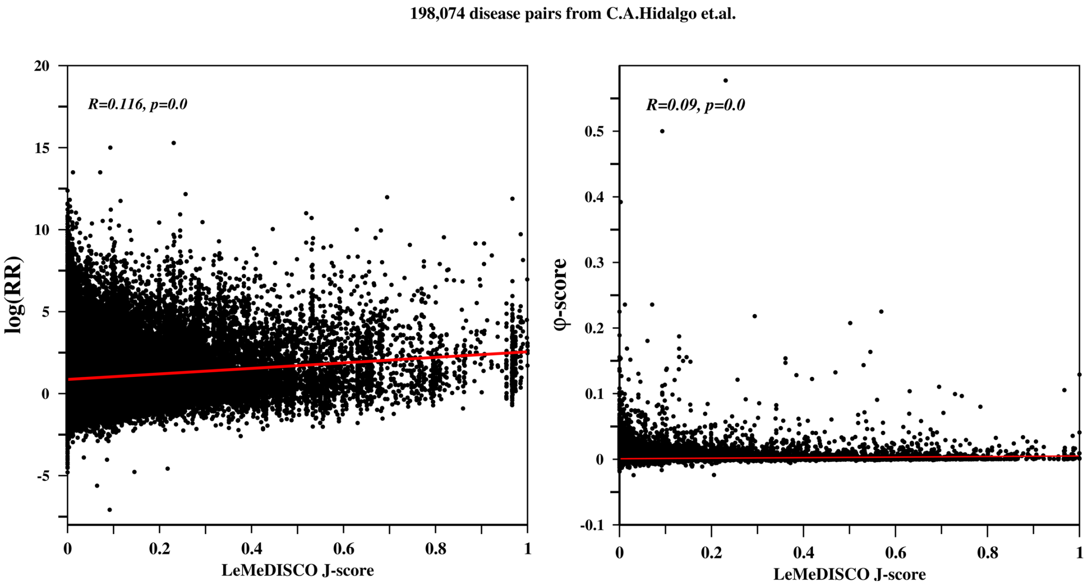

Supplementary Figure 4. Correlation between the J-score to the log(RR) score and φ-score for the 191,966 diseases from ref[1].

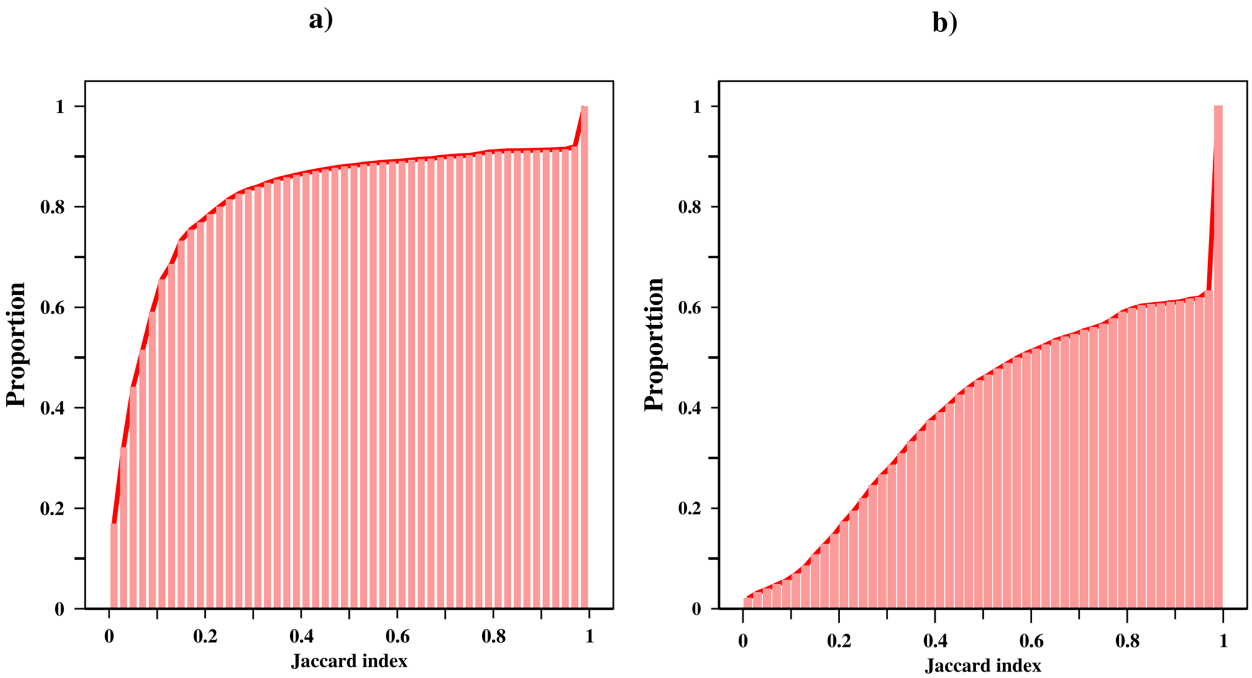

Supplementary Figure 5. Comorbidity distribution of J-score. a) cumulative distribution function (CDF) of the comorbidities with q-value < 0.05 and b) the top 100 comorbidities hierarchically ranked by J-score with q-value < 0.05.

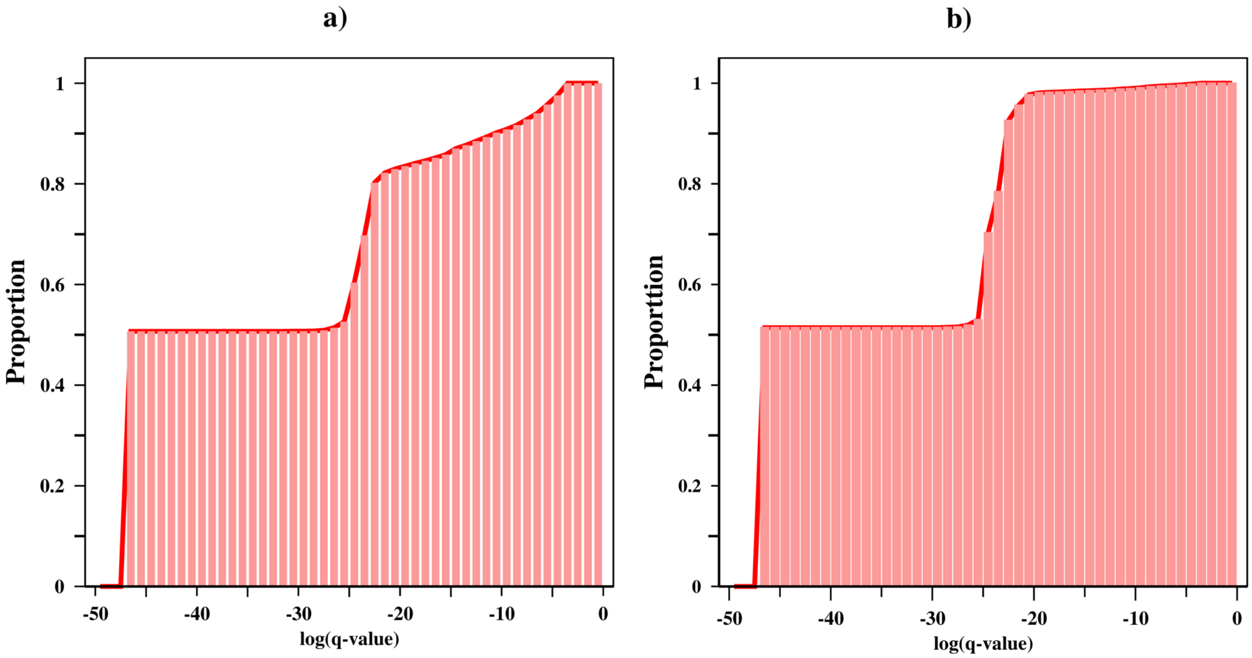

Supplementary Figure 6. CDFs of the comorbidities' log(q-values) with q-value < 0.05. a) top 100 most comorbidities hierarchically ranked by J-score with q-value < 0.05 b) q-values $<1.0\text{x}10^{-20}$ were set to$1.0\text{x}10^{-20}$.

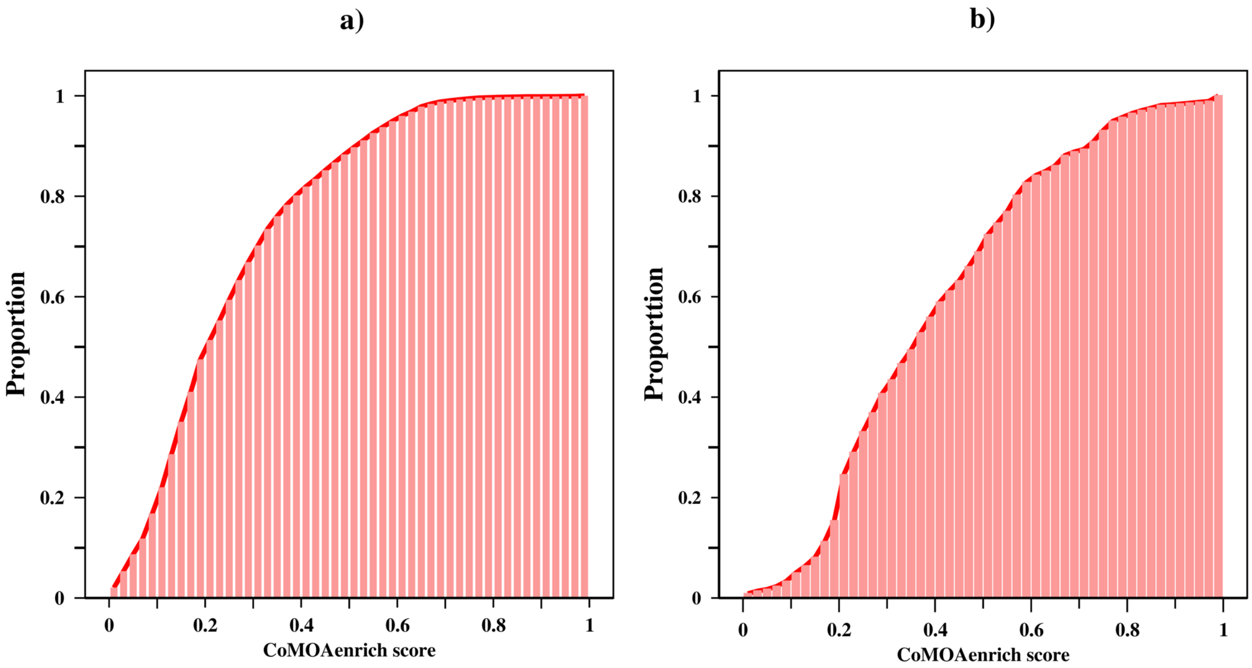

Supplementary Figure 7. CDFs of comorbidity enriched MOA protein vs. CoMOAenrich score. a) comorbidity enriched MOA protein vs. CoMOAenrich scores with CoMOAenrich score > 0.01 and b) the top the 100 comorbidity enriched MOA proteins hierarchically ranked by the CoMOAenrich score with CoMOAenrich score > 0.01.

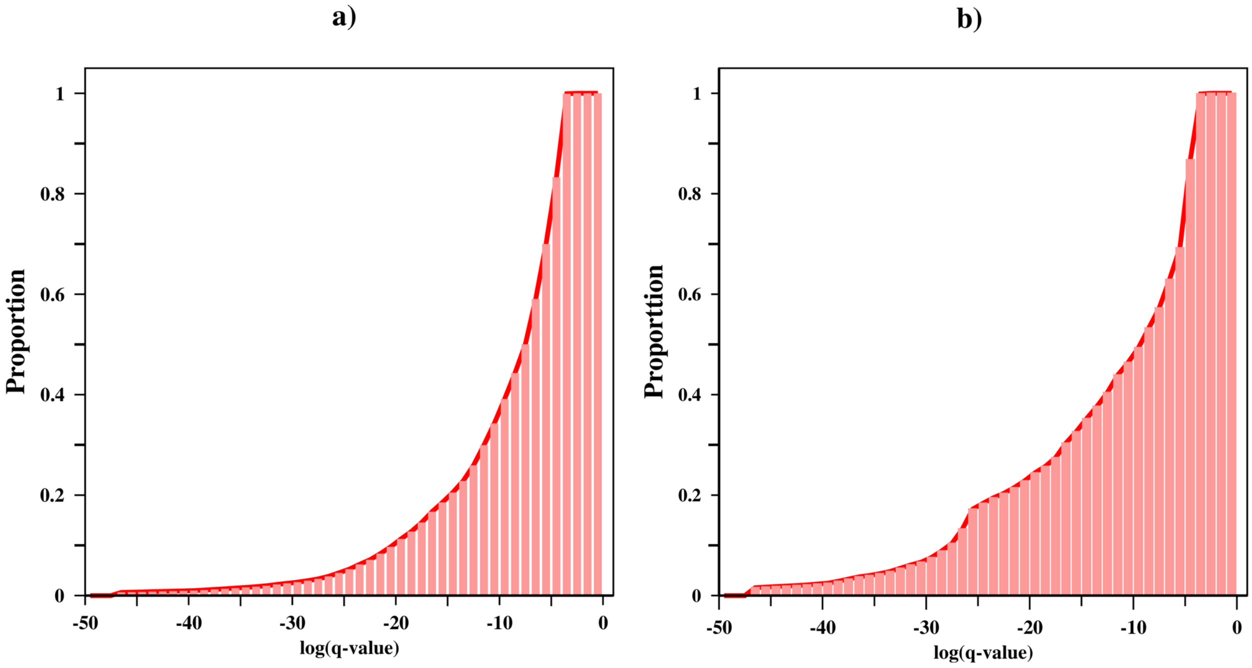

Supplementary Figure 8. CDFs of comorbidity enriched MOA protein vs. log(q-values). a) comorbidity enriched MOA protein vs. log(q-values) with CoMOAenrich score > 0.01 and b) top 100 comorbidity enriched MOA protein hierarchically ranked by the CoMOAenrich score with CoMOAenrich score > 0.01. q-values < $1.0x10^{-20}$ were set to$1.0x10^{-20}$.

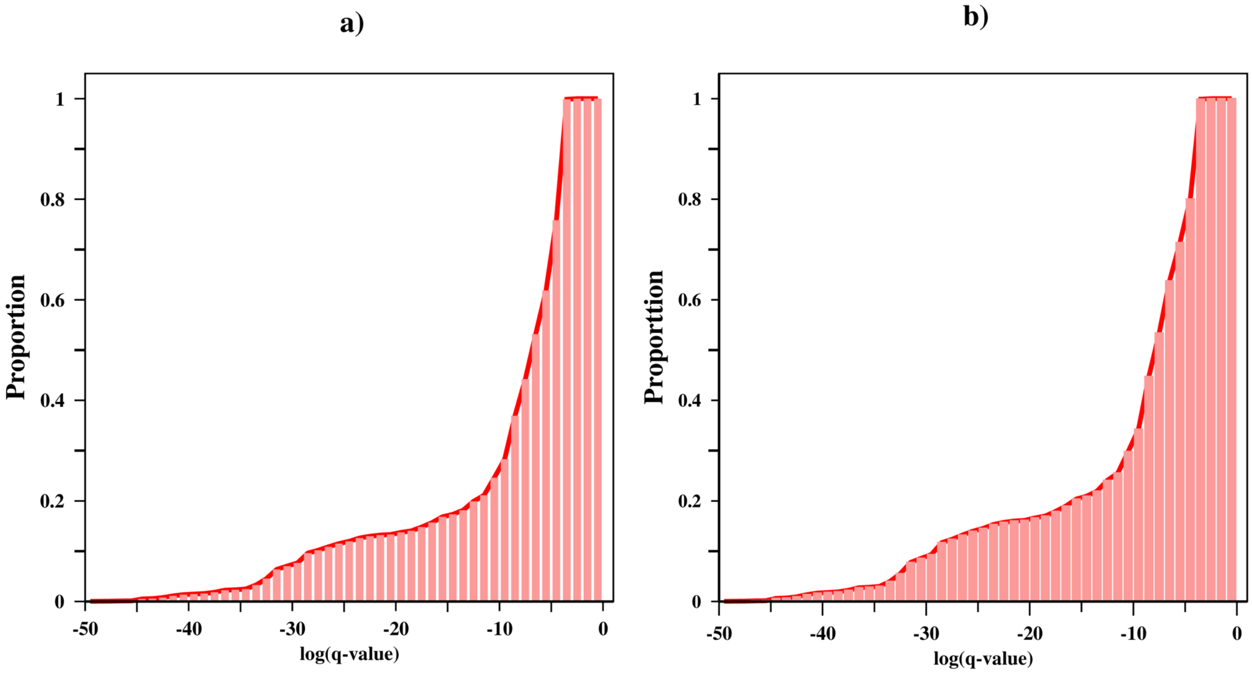

Supplementary Figure 9. CDFs of comorbidity enriched pathways vs. log(q-values). a) comorbidity enriched pathways vs. log(q-values) with q-value < 0.05, and b) top 100 comorbidity enriched pathways with q-value < 0.05 ranked by q-values in ascending order.

Supplementary Tables

Supplementary Table 1. Summary statistics of the MEDICASCY-driven LeMeDISCO scores for the comorbidities (excluding diagonals and redundant), comorbidity enriched MOA proteins, and pathways.

	**Count**[a]	**Mean**[b]	**Standard deviation**[c]	**Min**[d]	**Max**[d]
**Comorbidities**					
J-score					
q-value < 0.05	2,137,022	0.19	0.28	4.5e-04	1.0
Top 100 & q-value < 0.05	253,143	0.61	0.35	7.6e-04	1.0
q-value					
q-value < 0.05	2,137,022	1.1e-03	5.5e-03	0.0	0.05
Top 100 & q-value < 0.05	253,143	8.0e-05	1.4e-03	0.0	0.049
**Comorbidity-enriched MOA proteins**					
CoMOAenrich score					
CoMOAenrich score > 0.01	3,987,302	0.26	0.17	0.01	1.0
Top 100 & CoMOAenrich score > 0.01	301,787	0.41	0.21	0.01	1.0
q-value					
CoMOAenrich score > 0.01	3,987,302	7.6e-03	0.012	7.2e-66	0.05
Top 100 & CoMOAenrich score > 0.01	301,787	6.6e-03	0.011	1.4e-55	0.05
**Pathways**					
q-value					
q-value < 0.05	214,016	9.6e-03	0.013	4.2e-141	0.05
Top 100 & q-value < 0.05	176,336	7.5e-03	0.012	4.2e-141	0.05

[a]The count is the raw frequency of the comorbidities, MOA proteins, and pathways across diseases.
[b]The mean is the average of the respective scores/values across diseases.
[c]The standard deviation represents the variation of the scores/values across diseases.
[c]The min and max values are the absolute minimum and maximum score/value observed across diseases.

Supplementary Table 2. Literature evidence for the associations of randomly selected 20 diseases from 3608 diseases with Coronary artery disease and Ovarian cancer.

Randomly selected disease	Coronary artery disease	Ovarian cancer
Neuromyelitis optica	https://www.sciencedirect.com/science/article/pii/S0022510X21002240	https://www.ncbi.nlm.nih.gov/pmc/articles/PMC4941560/
Pancoast tumor	NO	https://pubmed.ncbi.nlm.nih.gov/11530393/
Empty sella syndrome	NO	NO
alexia	NO	NO
Subclavian artery aneurysm	https://ejrnm.springeropen.com/articles/10.1186/s43055-021-00621-y	NO
Vulvar disease	NO	NO
Subacute monocytic leukemia	NO	https://www.nejm.org/doi/full/10.1056/NEJM199001043220101
Constant exophthalmos	NO	NO
Colorado tick fever	https://www.ncbi.nlm.nih.gov/pmc/articles/PMC5930916/	NO
Tetanus neonatorum	NO	NO
Pleomorphic liposarcoma	NO	NO
Cheilitis	https://pubmed.ncbi.nlm.nih.gov/16276162/	NO
Renal infectious disease	NO	NO
Anterolateral myocardial infarction	https://www.hmpgloballearningnetwork.com/site/jic/article/7567	NO
Tropical sprue	https://openheart.bmj.com/content/5/1/e000668	NO
Autoimmune hemolytic anemia	https://pubmed.ncbi.nlm.nih.gov/34691339/	https://pubmed.ncbi.nlm.nih.gov/25918604/
Coronary stenosis	https://academic.oup.com/eurheartj/article/35/31/2069/2293101	NO
Foster-Kennedy syndrome	NO	https://morancore.utah.edu/section-05-neuro-ophthalmology/frontal-lobe-and-the-eye/
Anus neoplasm	NO	NO
Endocarditis	https://pubmed.ncbi.nlm.nih.gov/25362867/	https://www.ncbi.nlm.nih.gov/pmc/articles/PMC4941560/

Supplementary Table 3. Literature evidence for the associations of randomly selected 20 diseases after excluding LeMeDISCO predicted comorbid diseases with q-value<0.05 with Coronary artery disease and Ovarian cancer.

Comorbidities	Reference
**Coronary artery disease**	
laryngotracheitis	NO
adenoid hypertrophy	NO
autosomal recessive distal spinal muscular atrophy 1	NO
vertebral artery occlusion	https://academic.oup.com/qjmed/article/96/1/27/1526258
laryngostenosis	NO
Bordetella parapertussis whooping cough	https://www.nfid.org/2018/02/03/a-heart-healthy-lifestyle-includes-adult-vaccines/
cutaneous diphtheria	https://www.oatext.com/bacterial-cardiomyopathy-a-review-of-clinical-status-and-meta-analysis-of-diagnosis-and-clinical-management.php
tuberculous epididymitis	https://www.ncbi.nlm.nih.gov/pmc/articles/PMC3558260/
Huntington's disease	NO
paranasal sinus neoplasm	NO
frozen shoulder	NO
giant cell tumor	NO
ascaridiasis	https://www.ncbi.nlm.nih.gov/pmc/articles/PMC8387076/
Adenocarcinoma pancreas	NO
Brain stem glioma	NO
epidermolysis bullosa acquisita	NO
epidermolysis bullosa dystrophica	NO
kuru	NO
cataract	https://jamanetwork.com/journals/jamainternalmedicine/fullarticle/2511323
cholesteatoma	NO
**Ovarian cancer**	
Charcot-Marie-Tooth disease recessive intermediate B	NO
Brugada syndrome 8	NO
vaginal disease	https://www.nfcr.org/gynecologic-cancer-awareness-month/
episcleritis periodica fugax	NO
achromatopsia	NO
oral candidiasis	https://www.ncbi.nlm.nih.gov/pmc/articles/PMC4810127/
post-thrombotic syndrome	NO

chronic conjunctivitis	NO
lysosomal storage disease	NO
elephantiasis	NO
retinal detachment	NO
median neuropathy	NO
Salpingitis	https://pubmed.ncbi.nlm.nih.gov/8824371/
transient retinal arterial occlusion	NO
herpes zoster	https://www.ncbi.nlm.nih.gov/pmc/articles/PMC3593559/
vein disease	NO
Learning disability	NO
rectal prolapse	NO
toxocariasis	NO
hereditary choroidal atrophy	NO
