# Supplementary material for: LeMeDISCO is a computational method for large-scale prediction & molecular interpretation of disease comorbidity
Source: Commun Biol. 2022 Aug 25;5:870. doi: 10.1038/s42003-022-03816-9 (PMC9411158; doi:10.1038/s42003-022-03816-9)
Supplement: Supplementary file 2 — Description of Additional Supplementary Files [file 42003_2022_3816_MOESM2_ESM.pdf]

Description of Additional Supplementary Files

File name: Supplementary Data 1
Description: The source data for fig 1 a-e
